# Baicalein Alleviates Liver Oxidative Stress and Apoptosis Induced by High-Level Glucose through the Activation of the PERK/Nrf2 Signaling Pathway

**DOI:** 10.3390/molecules25030599

**Published:** 2020-01-30

**Authors:** Yuesheng Dong, Yan Xing, Jin Sun, Wenlong Sun, Yongbin Xu, Chunshan Quan

**Affiliations:** 1 School of Bioengineering, Dalian University of Technology, Dalian 116024, China; 2 Shandong Provincial Research Center for Bioinformatic Engineering and Technique, School of Life Sciences, Shandong University of Technology, Zibo 255049, China; 3 Key Laboratory of Biotechnology and Bioresources Utilization of Ministry of Education, Dalian Minzu University, Dalian 116024, China; 4 Department of Bioengineering, College of Life Science, Dalian Minzu University, Dalian 116600, China

**Keywords:** diabetes mellitus, baicalein, oxidative stress, apoptosis, liver, PERK/Nrf2 signaling pathway

## Abstract

Baicalein, a widely-distributed natural flavonoid, exhibits antioxidative activity in mice with type-2 diabetes. However, the underlying mechanisms remain partially elucidated. In this study, we investigated the effect of baicalein on protein kinase R-like ER kinase (PERK)/nuclear factor erythroid-2-related factor 2 (Nrf2) pathway for the alleviation of oxidative stress and apoptosis. Human liver HL-7702 cells were stimulated with 60.5 mM of glucose to induce oxidative stress and treated with baicalein. The apoptosis was determined by fluorescence microscopy and flow cytometry. The regulation of the PERK/Nrf2 pathway by baicalein was determined by immunoblotting in both HL-7702 cells and liver tissues from diabetic mice. We found that baicalein significantly alleviated the oxidative stress and apoptosis in HL-7702 cells stimulated with glucose. Mechanistic studies showed that baicalein downregulated PERK and upregulated Nrf2, two key proteins involved in endoplasmic reticulum stress, in both HL-7702 cells and liver tissues from diabetic mice receiving baicalein treatment. Furthermore, the subcellular localization of Nrf2 and the regulation of downstream proteins including heme oxygenase-1 and CCAAT-enhancer-binding protein homologous protein (CHOP) by baicalein were also investigated. Our results suggest that the regulation of the PERK/Nrf2 pathway is one of the mechanisms contributing to the bioactivities of baicalein to improve diabetes-associated complications.

## 1. Introduction

Diabetes mellitus (DM) is one of the most prevalent metabolic disorders in the 21st century, augmenting the health burden throughout the world. With the rapid development of the economy, the morbidity and mortality of diabetes has also been increasing dramatically worldwide in recent years. According to the data from the International Diabetes Federation, a total of 463 million adults are suffering from diabetes as of 2019, with 374 million at risk [1]. Furthermore, this number will reach 629 million by 2045 [1]. Type 2 diabetes mellitus (T2DM), caused by insulin resistance of the peripheral tissues, is the major form of diabetes, accounting for around 90% of the total incidence of DM [2,3]. The long-term hyperglycemic state in T2DM leads to the generation and accumulation of reactive oxygen species (ROS), which in turn leads to the endoplasmic reticulum stress (ERS) in the microvascular endothelial cells [4]. The ERS, if left uncontrolled, results in the apoptosis of the microvascular endothelium cells [5]. This will lead to the injury of the peripheral blood vessels, contributing to multiple microvascular complications of diabetes, including diabetic nephrosis, diabetic pedopathy, and diabetic cardiomyopathy [6,7].

The recent changes in the lifestyle, including excessive nutrition uptake and decreased amount of physical exercise, have increased the uptake and accumulation of glucose and exacerbated chronic oxidative stress. As the endoplasmic reticulum (ER) adaptive responses have not evolved sufficiently for the proper management of the oxidative stress encountered, maintenance or enhancement of proper ER function may contribute to the improvement of metabolic diseases, including insulin resistance and T2DM [5]. The ERS, which is accompanied by the activation of unfolded protein response (UPR), is mediated by protein kinase R-like ER kinase (PERK), an ER membrane-associated protein [8]. The ERS leads to the dissociation of the chaperone from PERK and then trigger UPR, which initially aims at rebuilding the cellular homeostasis by restoring the normal functions of the ER if the ERS is moderate and transient [9]. Nuclear erythroid-2-related factor (Nrf2) is a direct substrate and effector of PERK [10]. Upon oxidative stress, PERK directly phosphorylates Nrf2, leading to its dissociation from the cytoplasmic Nrf2/Keap1 complex [10]. The dissociated Nrf2 was then translocated into the nucleus to activate the expression of antioxidant response element (ARE)-dependent genes encoding a series of anti-oxidative and cytoprotective proteins, including heme oxygenase-1 (HO-1) [11,12]. HO-1 is an enzyme with cytoprotective activities including anti-inflammation and anti-apoptosis [6]. As shown in a recent study, the inhibition of Nrf2 exacerbated ERS and apoptosis, while the overexpression of Nrf2 reduced the level of CCAAT-enhancer-binding protein homologous protein (CHOP) and protected cells from apoptosis [13]. Thus, ERS is an attractive potential therapeutic target for the management of oxidative stress and insulin resistance. The regulation of PERK/Nrf2 pathway and the corresponding downstream signaling processes may contribute to the prevention and treatment of T2DM-associated complications. 

Baicalein, a flavonoid component of *Oroxylum indicum*, is also widely distributed in fruits, wine, tea, and vegetables [14,15]. As a polyphenolic compound, baicalein exhibits a variety of biological activities, including anti-oxidation, anti-inflammation, anti-hypertension, anti-bacteria, anti-cancer, and anti-virus [16,17,18]. Our previous studies showed that baicalein is an α-glycosidase inhibitor and exhibited a synergistic effect with acarbose in improving the postprandial glucose tolerance [19,20]. A subsequent in vivo study by our group showed that 4 weeks of baicalein treatment effectively improved hyperglycemia and regulated the gut microbiota in a rat T2DM model [21]. Besides, the combination of the extract of *Oroxylum indicum* seeds and baicalein with acarbose decreased the relative risk of progression from prediabetes to diabetes by 75% and 83.3% in vivo, respectively [22,23]. In a previous study, we established a mouse T2DM model by a high-fat diet and streptozotocin. The T2DM mice were then treated with 40 mg/kg/d and 160 mg/kg/d of baicalein to determine the anti-diabetic activity of baicalein [23]. The improvement of the histology in the hepatic tissues of diabetic mice by the treatment of baicalein at doses of 40 mg/kg/d and 160 mg/kg/d was also observed [23]. The anti-oxidation activity of baicalein in vivo was shown to be one of the main mechanisms for the control of diabetes and prediabetes, in which the regulation of the PERK/Nrf2 pathway might be involved [23].

In this study, we established an oxidative stress model in human liver HL-7702 cells by glucose stimulation to study the antioxidative activity of baicalein and its underlying molecular mechanisms. The antioxidative and anti-apoptosis activity of baicalein was determined by biochemical analysis, fluorescence microscopy, and flow cytometry. The effect of baicalein on the PERK/Nrf2 signaling pathway in HL-7702 cells and liver tissues of T2DM mice receiving baicalein was also investigated by immunoblotting and qRT-PCR.

## 2. Results

### 2.1. The Effect of Baicalein on the Viability of Glucose-Induced HL-7702 Cells

**Figure 1:**
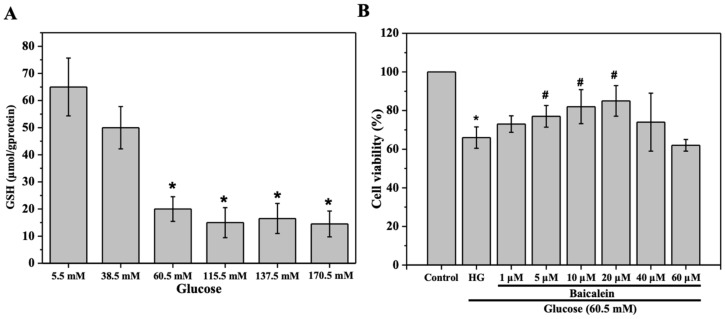
Determination of the doses of glucose and baicalein used in this study. (**A**) HL-7702 cells were grown in RPMI-1640 medium containing different concentrations of glucose for 24 h and the level of glutathione (GSH) in each group was determined. (**B**) HL-7702 cells were concomitantly treated with 60.5 mM of glucose and different doses of baicalein for 24 h and the viability of the cells was evaluated by 3-[4,5-dimethylthylthiazol-2-yl]-2,5-diphenyltetrazolium bromide (MTT) assay. The viability of cells exposed to 5.5 mM of glucose (control) was defined as 100%. The data were presented as the mean ± SD (*n* = 3). * *p* < 0.05 vs. control group; # *p* < 0.05 vs. glucose (HG) group.

We first determined the dose of glucose and baicalein to be used in the assays for the evaluation of the cytoprotective activity of baicalein. HL-7702 cells were stimulated with RPMI-1640 medium containing 5.5 mM, 38.5 mM, 60.5 mM, 115.5 mM, 137.5 mM, and 170.5 mM of glucose for 24 h, and the level of glutathione (GSH) was determined as a biomarker for the oxidative stress induced by glucose. As shown in Figure 1A, compared with the control group, in which the HL-7702 cells were grown in the medium containing 5.5 mM of glucose (with no external glucose added), the level of GSH in the cells grown in medium containing 38.5 mM of glucose was not significantly decreased (*p* > 0.05), whereas the level of GSH was significantly decreased in cells grown in medium containing 60.5 mM of glucose (*p* < 0.05). Higher doses of glucose also led to a significant decrease in the levels of GSH, while the data were not significantly different from that in cells induced by 60.5 mM of glucose (Figure 1A). Thus, 60.5 mM was chosen as the dose of glucose for the establishment of the oxidative stress model in HL-7702 cells, as it is the minimum dose of glucose required to generate significant oxidative stress.

The cells stimulated with 60.5 mM of glucose for 24 h were treated with a dose gradient of baicalein (1 μM, 5 μM, 10 μM, 20 μM, 40 μM, and 60 μM) to determine the dose of baicalein to be used. As shown in Figure 1B, exposure to 60.5 mM of glucose resulted in a significant decrease of cell viability (*p* < 0.05), whereas treatment with baicalein at doses of 1 μM to 20 μM alleviated the oxidative stress caused by glucose stimulation in a dose-dependent manner. Notably, treatment with baicalein at doses of 5 μM, 10 μM, and 20 μM resulted in a significant increase of the cell viability (*p* < 0.05) (Figure 1B). However, decreased cell viability was observed when the concentration of baicalein reached 40 μM. These data suggested that baicalein at doses lower than 40 μM exhibited a cytoprotective effect in HL-7702 cells with oxidative stress. Thus, 5 μM and 20 μM were selected as the doses of baicalein in the following studies.

### 2.2. The Effect of Baicalein on the Biomarkers of Oxidative Stress

**Figure 2:**
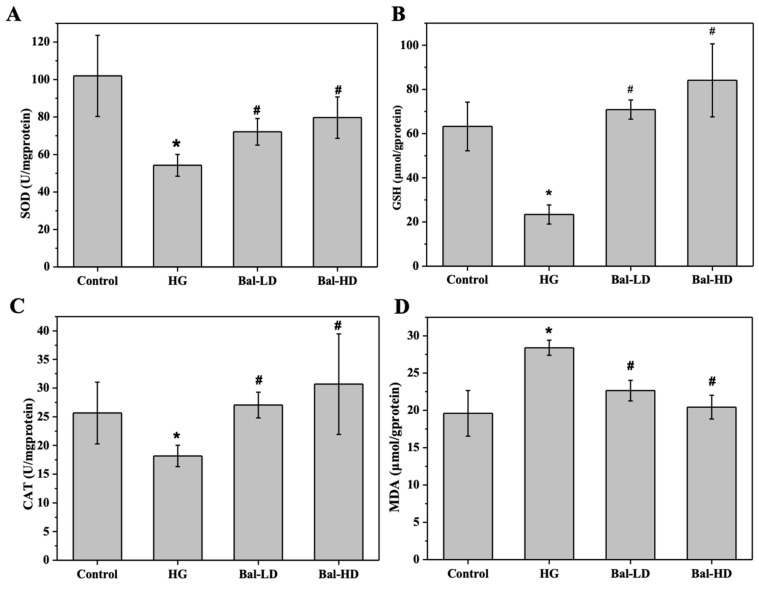
The effect of baicalein on the biomarkers of oxidative stress in HL-7702 cells stimulated with 60.5 mM of glucose. The levels of superoxide dismutase (SOD) (**A**), catalase (CAT) (**B**), GSH (**C**), and malondialdehyde (MDA) (**D**) were determined by the corresponding assay kit. The data were presented as the mean ± SD (*n* = 3). * *p* < 0.05 vs. control group; ^#^ *p* < 0.05 vs. HG group.

Growing evidence has shown that hyperglycemia induces reactive oxygen species (ROS), generating more oxidants, and less antioxidants. The level of biomarkers for oxidative stress, including malondialdehyde (MDA), glutathione (GSH), superoxide dismutase (SOD), and catalase (CAT), were determined. As shown in Figure 2, compared with the control group, the levels of SOD, CAT, and GSH were significantly decreased in HL-7702 cells exposed to 60.5 mM of glucose for 24 h (HG group), while a significant increase of the level of MDA was observed (*p* < 0.05). Treating HL-7702 cells with 5 μM (Bal-LD) and 20 μM (Bal-HD) of baicalein for 24 h significantly increased the activities of SOD, CAT, and GSH, and decreased the level of MDA (*p* < 0.05), suggesting that baicalein exhibited an antioxidative activity in HL-7702 cells with oxidative stress (Figure 2).

**Figure 3:**
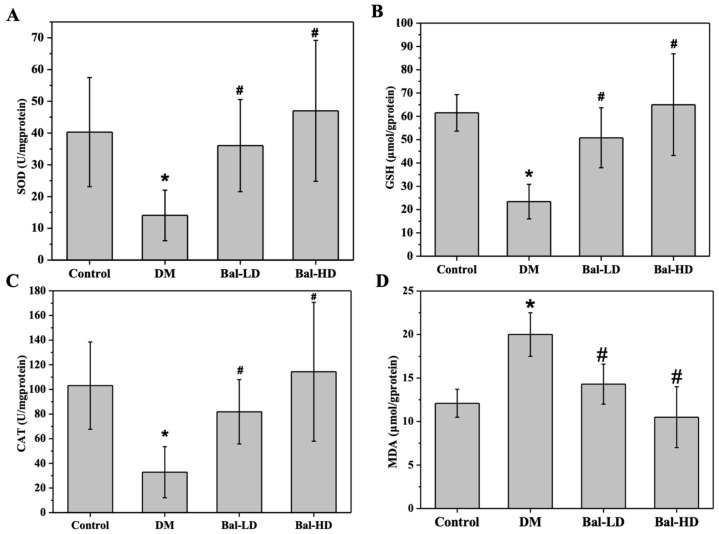
The effect of baicalein on the biomarkers of oxidative stress in the liver tissues of mice with type 2 diabetes mellitus (T2DM). The levels of SOD (**A**), CAT (**B**), GSH (**C**), and MDA (**D**) were determined by the corresponding assay kit. The data were presented as the mean ± SD (*n* = 3). * *p* < 0.05 vs. control group; ^#^ *p* < 0.05 vs. HG group.

The levels of biomarkers for oxidative stress in the liver tissues of T2DM mice receiving baicalein treatment for 8 weeks were determined and similar results were observed. The levels of SOD, CAT, and GSH were significantly decreased and the level of MDA was increased in DM group compared to the control group (*p* < 0.05). Treatment of the mice with 40 mg/kg/d (Bal-LD) and 160 mg/kg/d (Bal-HD) of baicalein for 8 weeks significantly improved the levels of SOD, CAT, and GSH, and decreased the level of MDA (*p* < 0.05) (Figure 3).

### 2.3. The Effect of Baicalein on the Level of Intracellular ROS in High-Glucose Induced HL-7702 Cells

**Figure 4:**
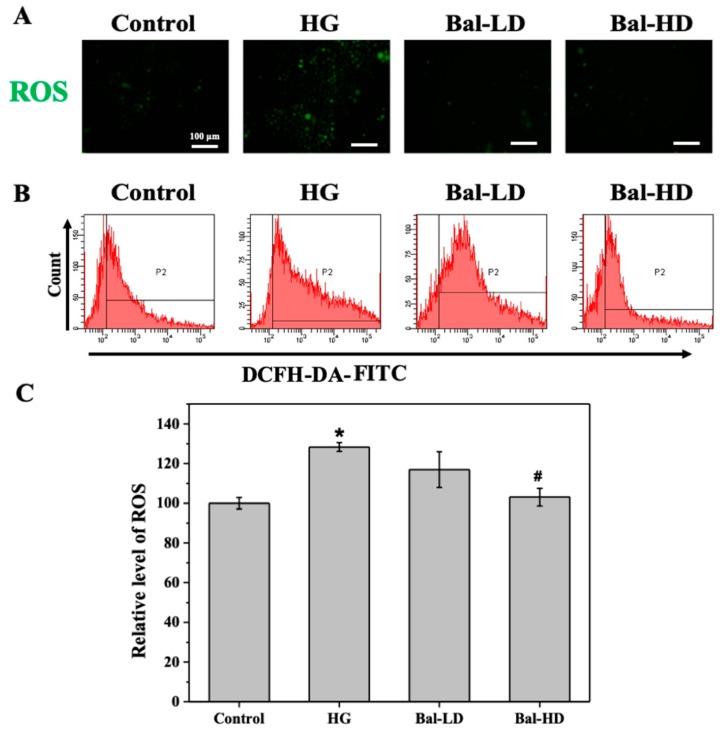
The effect of baicalein on the intracellular level of reactive oxygen species (ROS) in HL-7702 cells induced by 60.5 mM of glucose. HL-7702 cells were exposed to 5.5 mM of glucose (Control), 60.5 mM of glucose (HG), 60.5 mM of glucose with 5 μM of baicalein (Bal-LD), and 60.5 mM of glucose with 20 μM of baicalein (Bal-HD) for 24 h. The level of ROS was assessed by fluorescence microscopy and flow cytometry. (**A**) Images indicating the level of ROS (green fluorescence) in HL-7702 cells. (**B**) The representative data of ROS-positive cells determined by flow cytometry. (**C**) The relative level of ROS in cells of each group revealed by flow cytometry. The data are shown as the mean ± SD (*n* = 3). * *p* < 0.05 vs. control group; ^#^ *p* < 0.05 vs. HG group.

We then investigated the level of ROS in response to glucose stimulation and the effect of baicalein on the level of ROS in HL-7702 cells. As shown in Figure 4A, few cells were labeled with 2,7-dichlorodihydrofluorescein-diacetate (DCFH-DA) in the control group, indicating a low level of intracellular ROS. In contrast, the numbers of cells labeled increased dramatically in the group of cells stimulated with 60.5 mM of glucose (HG group), suggesting that glucose treatment increased the level of ROS in HL-7702 cells. Treatment of HL-7702 cells with both 5 μM and 20 μM of baicalein alleviated the increased level of ROS induced by 60.5 mM of glucose (Figure 4A). Flow cytometry was further performed to evaluate the level of ROS in the whole cell population (Figure 4B). According to the results, stimulation with 60.5 mM of glucose resulted in a 28% increase in the intracellular level of ROS of HL-7702 cells as compared with the control group. Treatment with baicalein alleviated the increase of ROS (Figure 4C). The level of ROS in cells treated with 20 μM of baicalein was significantly decreased compared to the HG group and was comparable to the control group (Figure 4B,C). These data indicated that the level of intracellular ROS elevated by glucose stimulation was effectively controlled by the treatment of baicalein.

### 2.4. The Effect of Baicalein on the Apoptosis of High Glucose-Induced HL-7702 Cells

**Figure 5:**
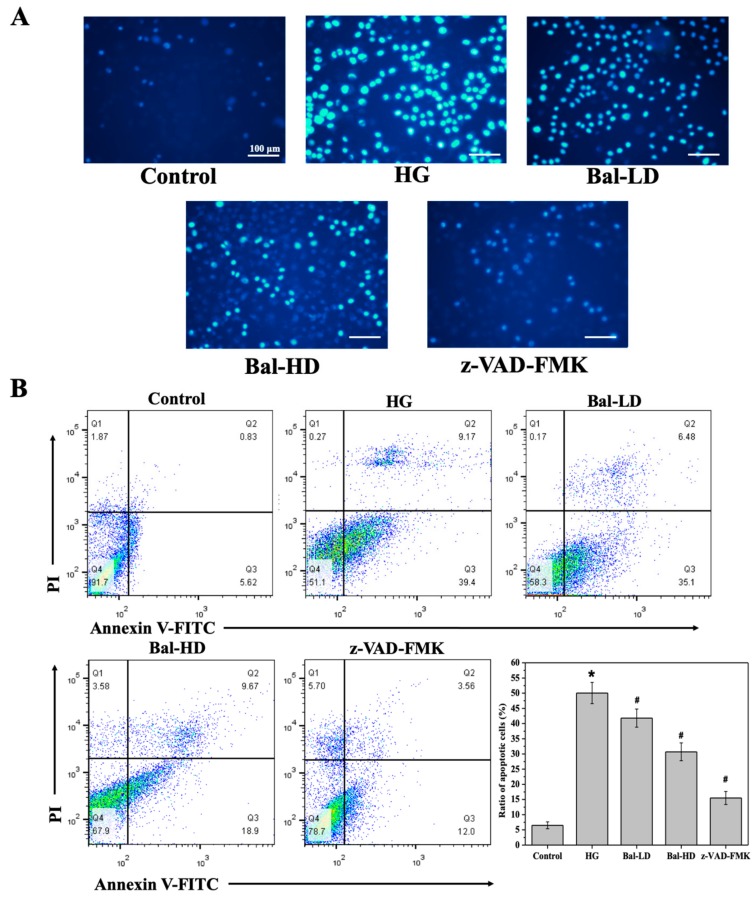
The effect of baicalein on the apoptosis of HL-7702 cells induced by 60.5 mM of glucose. HL-7702 cells were exposed to 5.5 mM of glucose (Control), 60.5 mM of glucose (HG), 60.5 mM of glucose with 5 μM of baicalein (Bal-LD), 60.5 mM of glucose with 20 μM of baicalein (Bal-HD), and 60.5 mM of glucose with a pan-caspase inhibitor (z-VAD-FMK) for 24 h. The level of apoptosis in each group was determined. (**A**) Fluorescence images showed the apoptosis levels in the HL-7702 cells stained with Hoechst 33824. Apoptotic cells with strong blue fluorescence were observed. (**B**) Quantitative analysis of the level of apoptosis in each group. The cells were stained with AnnexinV-FITC/PI and the apoptosis of the cells in each group was evaluated by flow cytometry. The data are represented as the mean ± SD (*n* = 3). * *p* < 0.05 vs. control group; # *p* < 0.05 vs. HG group.

Hoechst 33824 staining was performed to investigate the effect of baicalein on the apoptosis of HL-7702 cells induced by 60.5 mM of glucose [24]. In apoptotic cells, the condensed chromatin was stained with Hoechst 33824 and elicited strong blue fluorescence upon excitation. As shown in Figure 5A, few normal HL-7702 cells were stained by Hoechst 33824, suggesting a low baseline level of apoptosis. However, more glucose-stimulated cells were stained by Hoechst 33824, with a strong fluorescence observed, which was effectively reversed by the apoptosis inhibitor z-VAD-FMK (Figure 5A). These data indicated that apoptosis was induced in cells stimulated with glucose. Treatment with baicalein suppressed apoptosis induced by 60.5 mM of glucose, as is shown by the decreased staining of cells treated with 5 μM and 20 μM of baicalein (Figure 5A). The quantitative analysis by flow cytometry suggested that treatment with 5 μM and 20 μM of baicalein decreased the proportion of apoptotic cells by 20% and 40%, respectively (Figure 5B). 

### 2.5. The Effect of Baicalein on the Activation of the PERK/Nrf2 Pathway In Vitro and In Vivo

**Figure 6:**
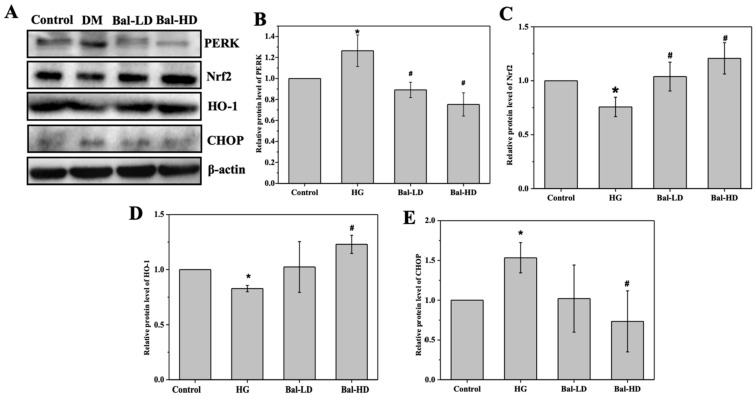
The effect of baicalein on the level of PERK, Nrf2, HO-1, and CHOP in HL-7702 cells. HL-7702 cells were incubated with 60.5 mM of glucose and baicalein (5 μM and 20 μM) for 24 h. The levels of target protein were determined by immunoblotting. (**A**) Representative immunoblots of the proteins including PERK, Nrf2, HO-1, and CHOP. (**B**–**E**) Quantitative analysis and statistical analysis of the level of PERK, Nrf2, HO-1, and CHOP relative to the control group, respectively. The data are represented as the mean ± SD (*n* = 3). * *p* < 0.05 vs. control group; ^#^ *p* < 0.05 vs. HG group.

We further investigated the effect of baicalein on the PERK/Nrf2 signaling pathway to study the molecular mechanism of the antioxidative and anti-apoptosis activity of baicalein. The results of immunoblotting using HL-7702 cell lysate showed that the levels of Nrf2 and HO-1 were decreased in HL-7702 cells exposed to 60.5 mM of glucose (HG group) and that the levels of PERK and CHOP were increased (Figure 6A). Notably, this trend was significantly reversed by 20 μM of baicalein. The protein level of PERK increased by 26.5% in the HG group compared with the control group and was decreased by 29.5% and 40.5% upon the treatment with 5 μM of baicalein (Bal-LD) and 20 μM of baicalein (Bal-HD), respectively (Figure 6A,B). The protein level of CHOP was increased by 50.6% in the HG group as compared to the control group and decreased by 31.0% and 50.0% in the Bal-LD group and Bal-HD group, respectively (Figure 6A,E). The protein level of Nrf2 was decreased by 24.2% in the HG group compared with the control group and was increased by 27.0% and 37.2% in the Bal-LD group and Bal-HD group, respectively (Figure 6A,C). The protein level of HO-1 was decreased by 17.2% in the HG group compared with the control group and was increased by 19.2% and 32.7% in the Bal-LD group and Bal-HD group, respectively (Figure 6A,D).

**Figure 7:**
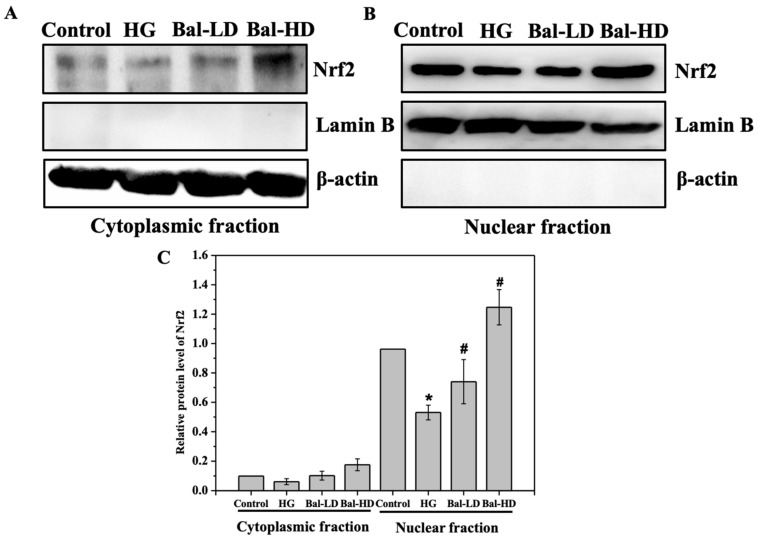
The subcellular localization of Nrf2 in HL-7702 cells. HL-7702 cells were stimulated with 60.5 mM of glucose and incubated with baicalein (5 μM and 20 μM) for 24 h. (**A**, **B**) The levels of Nrf2 in the cytoplasmic fraction and in the nuclear fraction were determined by immunoblotting. (**C**) The level of Nrf2 in each group relative to the control was quantified. The data are represented as the mean ± SD (*n* = 3). * *p* < 0.05 vs. control group; # *p* < 0.05 vs. HG group.

We then set out to determine the subcellular localization of Nrf2 in HL-7702 cells upon glucose stimulation and baicalein treatment. As shown in Figure 7, the majority of the Nrf2 was identified in the nuclear fraction of HL-7702 cell lysate. Upon baicalein treatment, the protein level of Nrf2 was increased in both cytoplasmic fraction and nuclear fraction (Figure 7). Especially, the protein level of Nrf2 was significantly increased in the nuclear fraction, with a 30% increase in HL-7702 cells treated with 20 μM of baicalein (Figure 7C). This result suggests that the majority of Nrf2 protein is released from the Nrf2/Keap1 complex and translocated to the nucleus upon baicalein treatment to initiate the transcription of the downstream antioxidative genes, which is consistent to the role of Nrf2 as a master transcription factor playing major roles in the defense against oxidative stress.

**Figure 8:**
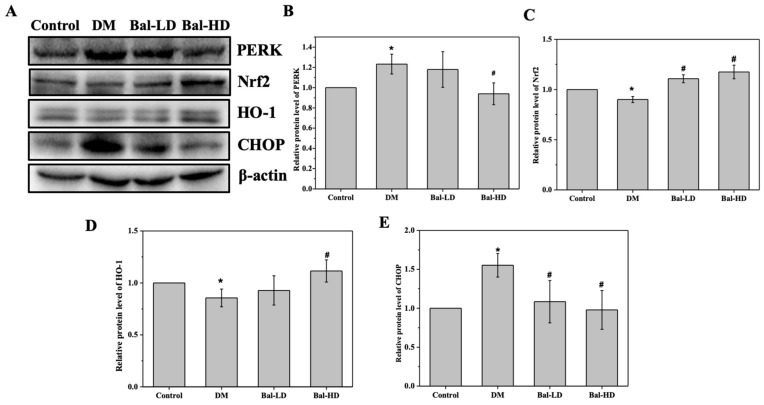
The effect of baicalein on the protein level of PERK, Nrf2, HO-1, and CHOP in liver tissues of T2DM mice treated with baicalein. The liver tissues from normal mice (control group), T2DM mice (DM group), T2DM mice treated with 40 mg/kg/d of baicalein (Bal-LD group) and T2DM mice treated with 160 mg/kg/d of baicalein (Bal-HD group) were homogenized. The levels of the target proteins were determined by immunoblotting. (**A**) Representative immunoblot of the proteins including PERK, Nrf2, HO-1, and CHOP. (**B**–**E**) Quantitative analysis and the statistical analysis of the protein levels of PERK, Nrf2, HO-1, and CHOP relative to the control group. Values are represented as the mean ± SD (*n* = 3). * *p* < 0.05 vs. control group; # *p* < 0.05 vs. DM group.

In a previous study, we investigated the hypoglycemic role of baicalein and the anti-oxidative activity of baicalein in a mouse T2DM model [23]. Upon 8-week of baicalein treatment, the glucose tolerance of the mice was significantly improved [23]. We further collected the liver tissues from the T2DM mice that received different doses of baicalein for 8 weeks to investigate the role of baicalein on the oxidative stress and the underlying molecular mechanisms. Consistent with the results revealed in HL-7702 cells, the protein levels of Nrf2 and HO-1 were decreased and the protein levels of PERK and CHOP were increased in T2DM mice (Figure 8). The levels of the proteins were significantly reversed by 160 mg/kg/d of baicalein in a dose-dependent manner. The protein level of PERK increased by 23.2% in T2DM mice (DM group) as compared to the control group, which was decreased by 4.3% and 23.8% in baicalein 40 mg/kg/d (Bal-LD) group and baicalein 160 mg/kg/d (Bal-HD) group respectively (Figure 8A,B). The protein level of CHOP increased by 45.3% in the DM group as compared with the control group, which was decreased by 25.4% and 32.6% in the Bal-LD group and Bal-HD group, respectively (Figure 8A,F). The protein level of Nrf2 was decreased by 5.9% in the DM group as compared with the control group, which was increased by 15.1% and 20.0% in the Bal-LD group and Bal-HD group, respectively (Figure 8A,C). The protein level of HO-1 was decreased by 14.4% in the DM group as compared with the control group, which was increased by 7.7% and 23.3% in the Bal-LD group and Bal-HD group, respectively (Figure 8A,D).

**Figure 9:**
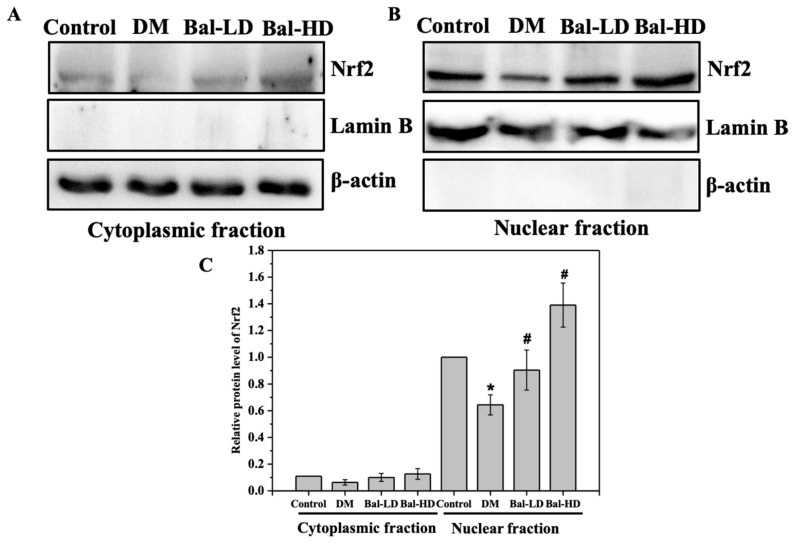
The subcellular localization of Nrf2 in the liver tissue of T2DM mice treated with baicalein. The liver tissues from normal mice (control group), mice model of T2DM (DM group), T2DM mice treated with 40 mg/kg/d of baicalein (Bal-LD group), and T2DM mice treated with 160 mg/kg/d of baicalein (Bal-HD group) were homogenized. The levels of the target proteins were determined by immunoblotting. Representative immunoblot of the Nrf2 in the cytoplasmic fraction (**A**) and nuclear fraction (**B**). Quantitative analysis and the statistical analysis of the protein levels of Nrf2 relative to the control group (**C**). Values are represented as the mean ± SD (*n* = 3). * *p* < 0.05 vs. control group; # *p* < 0.05 vs. DM group.

We also determined the subcellular localization of Nrf2 in liver tissues of T2DM mice treated with baicalein. As shown in Figure 9, most of the Nrf2 was identified in the nuclear fraction of liver tissue homogenate (Figure 9A,B). Especially, the protein level of Nrf2 was significantly increased in the nuclear fraction, with a 28% increase in the liver tissue of T2DM mice treated with 160 mg/kg/d baicalein (Figure 9C). These results confirmed the role of baicalein on the activation of Nrf2 in hepatocytes of T2DM mice receiving baicalein at a dose of 160 mg/kg/d.

**Figure 10:**
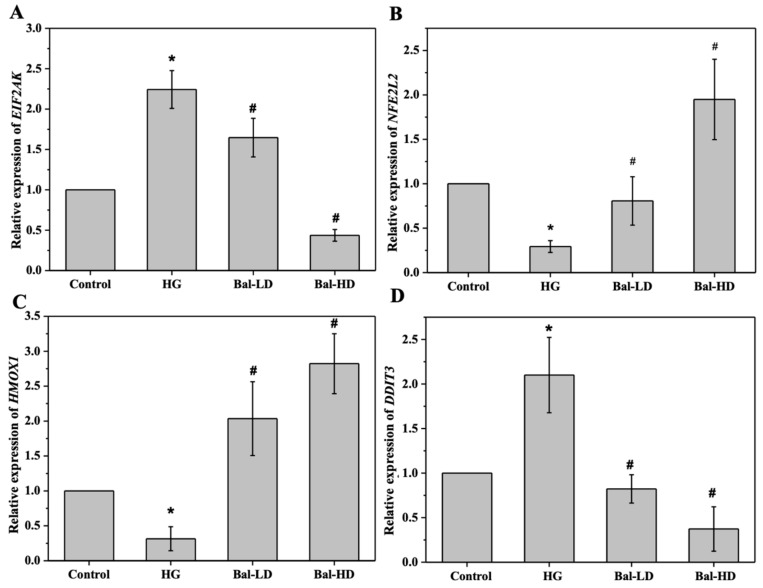
The effect of baicalein on the mRNA levels of *EIF2AK3*, *NFE2L2*, *HMOX1*, and *DDIT3*. HL-7702 cells were stimulated with 60.5 mM of glucose and treated with 5 μM and 20 μM of baicalein for 24 h. The mRNA levels of *EIF2AK* (encoding PERK) (**A**), *NFE2L2* (encoding Nrf2) (**B**), *HMOX1* (encoding HO-1) (**C**), and *DDIT3* (encoding CHOP) (**D**) in each group relative to the control was determined by qRT-PCR. The data are represented as the mean ± SD (*n* = 3). * *p* < 0.05 vs. control group; ^#^ *p* < 0.05 vs. HG group.

To further investigate the underlying mechanism of the regulation of the key proteins in the PERK/Nrf2 signaling pathway by baicalein, we investigated the expression of the genes encoding these proteins by qRT-PCR. As shown in Figure 10, HL-7702 cells co-treated with baicalein and 60.5 mM of glucose showed significantly increased mRNA levels of *NFE2L2*, which encodes Nrf2, and Nrf2-dependent ARE gene *HMOX1*, which encodes HO-1, compared to those treated with 60.5 mM of glucose alone (*p* < 0.05) (Figure 10B,D). In contrast, the mRNA level of *EIF2AK3*, which encodes PERK, and *DDIT3*, which encodes CHOP, were significantly decreased in HL-7702 cells following the treatment of baicalein (*p* < 0.05) (Figure 10A,C), with a dose-dependent pattern observed. These data suggest that baicalein regulates the level of key proteins in the PERK/Nrf2 pathway by regulating the expression of their encoding genes.

## 3. Discussion

Baicalein, also known as 5,6,7-trihydroxyflavone, is a major active component of *Oroxylum indicum*, *Scutellaria baicalensis*, and *Tuber aestivum*, which are consumed as food, tea, or dietary supplements in China, the United States, Southeast Asia, and in Europe [25]. Both in vitro and in vivo studies of baicalein suggest that the anti-oxidation activity of this flavonoid were one of the mechanisms contributing to the treatment and prevention of diabetes [23]. Previous studies have also revealed the effect and molecular mechanisms of baicalein on oxidative stress. Baicalein can restore the damage of cell organelles caused by oxidative stress by decreasing the expression of phospho-H2A.X, DNA tail formation, and the formation of thiobarbituric acid reactive substances [26]. Meanwhile, baicalein inhibited protein oxidation through protein carbonyl formation [26]. Baicalein was shown to upregulate the expression of antioxidant enzymes, including glutathione peroxidase and SOD, in rats with T2DM [27]. Baicalein supplementation was shown to control the function of membrane permeability transition pore (MPTP) to prevent DNA damage induced by ROS in the lungs during B(a)P-induced pulmonary carcinogenesis [28]. These studies showed that baicalein exhibits antioxidative activity through multiple mechanisms. 

Accumulating evidence has indicated that natural chemicals regulate ER stress via the PERK/Nrf2 signaling pathway. Özcan et al. showed that treating obese and diabetic mice with 4-phenyl butyric acid and taurine-conjugated ursodeoxycholic acid alleviated ER stress in cells and animals through the activation of the PERK/Nrf2 pathway [5]. The antioxidative activity and Nrf2-activating activity of multiple flavonoids were reported in previous studies [6,29,30]. For instance, mangiferin, a natural C-glucoside xanthone, enhanced the function of Glo-1 by inducing the activation of the Nrf2/ARE signaling pathway in central neurons stimulated with high-level of glucose [29]. Myricitrin, a botanical flavone identified in multiple medicinal herbs, alleviated the apoptosis of H9c2 cardiomyocytes through the activation of the Akt-Nrf2 signaling pathway [6]. Another previous research showed that baicalein could restore both the protein level and the enzymatic activity of manganese superoxide dismutase (MnSOD) and its regulator Nrf2, which were abolished by H_2_O_2_ treatment [30]. The molecular mechanisms of the antioxidative and anti-apoptosis activities of the natural products are still under investigation. These natural products are all flavonoids, which are derivatives of the 2-phenyl chromone. A common structural characteristic of these bioactive flavonoids is the multiple phenolic hydroxyl groups, which are reductive and confer the biological activities of these compounds, including anti-apoptosis and anti-oxidation [31]. In this study, we found that baicalein regulated the PERK/Nrf2 signaling pathway in glucose-stimulated HL-7702 cells for the first time. Our research also confirmed the regulatory effect of baicalein on its effector Nrf2 and the oxidative stress-related protein, HO-1, suggesting that the regulation of PERK/Nrf2 is a potential mechanism for the antioxidative activity of baicalein. Baicalein also exhibited protection against apoptosis in HL-7702 cells. Apoptosis-related proteins, including CHOP, were also downregulated following the treatment of baicalein. Recently, a study demonstrated that the overexpression of Nrf2 significantly reduced CHOP-dependent apoptosis in retinal pigment epithelium cells [13]. In contrast, knockdown of *NFE2L2*, the coding gene of Nrf2, exacerbated the apoptosis induced by cigarette smoke extract, suggesting that CHOP is required for Nrf2 upregulation [13]. Thus, the regulation of PERK/Nrf2 by baicalein contributes to the protection of hepatic cells from apoptosis. The detailed mechanism of the effect of baicalein on PERK still warrants in-depth investigation in the future.

The regulation of the PERK/Nfr2 pathway was also reported to contribute to the improvement of insulin resistance. PERK was involved in at least two distinct signaling processes: (1) The inhibition of PERK can improve the insulin sensitivity by promoting the activities of forkhead box protein O (FOXO) and Akt [32]; (2) PERK can generate phosphatidic acid by direct phosphorylation of diacylglycerol, and then trigger Akt activation [8]. Besides, the induction of Nrf2 was reported to protect pancreatic islets by activating the expression of antioxidant genes [8]. The induction of Nrf2 also increased the expression of energy consumption-related genes in skeletal muscle and decreased that of gluconeogenesis-related genes in the liver, thereby leading to the improvement of insulin resistance [33]. According to our previous in vivo study, administration of baicalein led to an increased level of hepatic glycogen and upregulated phosphorylation of Akt1, consequently improving the insulin resistance and decreasing the expression of SOCS3 [22]. Thus, the regulation of the PERK/Nrf2 pathway by baicalein verified in this study, together with the regulation of SOCS3 and other mechanisms, contributes to the improvement of insulin resistance for the prevention and treatment of diabetes. 

## 4. Materials and Methods

### 4.1. Materials and Reagents

All materials for cell culture were purchased from Corning Co. (Corning, NY, USA). Baicalein was purchased from Chengdu Mansite Biotechnology Institute (Chengdu, China). 3-[4,5-dimethylthylthiazol-2-yl]-2,5-diphenyltetrazolium bromide (MTT) was purchased from Sigma Co. (St. Louis, MO, USA). Bicinchoninic acid (BCA) protein assay kit, 4’,6-diamidino-2-phenylindole (DAPI), and protease inhibitor cocktail were purchased from Solarbio Co., Ltd (Beijing, China). Annexin-FITC apoptosis detection kit and assay kits for total ROS, MDA, GSH, SOD, and CAT were purchased from Nanjing Jiancheng Bioengineering Institute (Nanjing, China). Cytoplasmic protein and nuclear protein isolation kit was purchased from WanleiBio Co., Ltd (Shenyang, China). TRIzol reagent, cDNA synthesis kit and SYBR Green PCR Master Mix were purchased from TaKaRa Biotechnology Co. (Dalian, China). Anti-Nrf2, anti-HO-1, anti-CHOP, and anti-Lamin B monoclonal antibodies were purchased from Proteintech Co., Ltd. (Wuhan, China). Anti-PERK antibody and goat anti-rabbit IgG conjugated with horseradish peroxidase were purchased from Bioss Co., Ltd. (Beijing, China).

### 4.2. Cell Culture and Treatment

HL-7702, a human liver cell line, was kindly provided by Prof. Yixin Ren from the College of Basic Medicine, Dalian Medical University (Dalian, China). The cells were cultured in Roswell Park Memorial Institute (RPMI)-1640 medium supplemented with 10% (*v*/*v*) fetal bovine serum, 100 U/L penicillin, and 100 mg/L streptomycin. The cells were maintained in a humidified incubator with 5% humidified CO_2_ atmosphere at 37 °C. Baicalein was dissolved in DMSO as a stock solution and diluted with RPMI 1640 medium when used. The concentration of DMSO was 0.1% (*v*/*v*) in all groups. For all assays, an equal number of cells were seeded into each well of 6-well plates or 96-well plates according to the experimental design and were grown for 24 h to reach 70–80% confluency before each assay.

### 4.3. Assays of Cell Viability and Oxidative Stress

The cell viability was determined by MTT assay. HL-7702 cells were seeded at a density of 3 × 10^3^ cells per well in 96-well plates. After a 24-h incubation, the cells were incubated with complete medium (control group), complete medium with 60.5 mM of glucose (HG group), and complete medium with 60.5 mM of glucose supplemented with 1 μM, 5 μM, 10 μM, 20 μM, 40 μM, and 60 μM of baicalein, respectively. After incubation for 24 h, the medium was removed, and the cells were stained with MTT (5 mg/mL) at 37 °C for 4 h. Subsequently, the medium was removed and DMSO (150 μL) was added to each well to dissolve the formazan crystals. The absorbance of each well was determined at 570 nm by a Thermo Scientific Varioskan^®^ Flash microplate reader (Thermo Fisher Scientific, Inc., Waltham, MA, USA). The viability of the HL-7702 cells was expressed as the ratio to the viability of the cells in the control group.

After treatment with glucose and baicalein, the levels of GSH, MDA, SOD, and CAT of cells in each group were determined with the corresponding detection kit according to the manufacturer’s instructions (Nanjing Jiancheng Biotechnology Institute, Nanjing, China).

### 4.4. Detection of Intracellular Level of ROS in HL-7702 Cells

The level of intracellular ROS in HL-7702 cells of each group was determined by a commercially available ROS detection kit according to the manufacturer’s brochure (Nanjing Jiancheng Biotechnology Institute, Nanjing, China). Briefly, HL-7702 cells were seeded on coverslips in 24-well plates with a density of 1 × 10^4^ and incubated at 37 °C for 24 h. After treatment by 60.5 mM of glucose and various doses of baicalein, the cells were washed twice with PBS. The cells were then incubated with ROS detection solution and cell nucleus detection solution at 37 °C in dark for 30 min, respectively. The cells were subsequently harvested, suspended in PBS with an approximate density of 1 × 10^6^ cells/mL and observed by an IX83 inverted fluorescence microscope (Olympus Co., Tokyo, Japan). The intracellular ROS was labeled by DCFH-DA, and the nuclei were labeled by DAPI. These cells were further analyzed by flow cytometry to quantify the intracellular level of ROS (BD Biosciences Co., San Jose, CA, USA). 

### 4.5. Analysis of Cell Apoptosis

The cells in each group were stained by Hoechst 33824 (Majorbio Co. Ltd, Shenyang, China) and the blue fluorescence of the nuclei was observed by an IX83 inverted fluorescence microscope (Olympus Co., Tokyo, Japan). The apoptosis of the cells in each group was then quantitively evaluated by flow cytometry with Annexin V-FITC/PI apoptosis detection kit according to the manufacturer’s brochure (Nanjing Jiancheng Biotechnology Institute, Nanjing, China). In brief, the cells were harvested, washed twice with cold PBS, incubated with Annexin V-FITC and PI working solution in binding buffer for 10 min in dark at room temperature. The intracellular fluorescence of the cells in each group was measured by flow cytometry with a FACS Calibur flow cytometer (BD Biosciences Co., San Jose, CA, USA).

### 4.6. Immunoblotting of the Key Proteins in PERK/Nrf2 Signaling Pathway

The cells in each group were harvested, washed with PBS, and lysed by cell lysis buffer containing protease inhibitors and 1 mM phenylmethylsulfonyl fluoride (PMSF). The cell lysate was centrifuged at 11,600 *g* for 20 min at 4 °C to remove the cell debris, and the supernatant containing the total protein extract was collected. The protein concentration of each sample was determined by the BCA protein assay. An equal amount of protein was separated by SDS-PAGE and then transferred onto PVDF membranes (MilliporeSigma Co., Burlington, MA, USA) in Tris-glycine buffer at 100 mA for 2 h. The membranes were blocked with 5% (*w*/*v*) nonfat milk in Tris-buffered saline containing 0.1% (*v*/*v*) Tween-20 (TBST) for 2 h. The membrane was then incubated with the appropriate primary antibodies at a dilution ratio of 1:2000, including anti-PERK, anti-Nrf2, anti-HO-1, anti-CHOP, and anti-β-actin at 4 °C overnight. After three washes with TBST, the membrane was incubated with horseradish-conjugated goat-anti-rabbit secondary antibody at a dilution ratio of 1:3000 for 1 h at room temperature in TBST with 1% nonfat milk. After three additional washes with TBST, the membrane was developed by ECL reagent. The intensity of the bands was quantified by Image Lab Software (Bio-Rad Laboratories, Inc., Hercules, CA, USA).

### 4.7. Obtention and Processing of the Liver Tissues from T2DM Mice

Liver tissues of T2DM mice treated with baicalein were obtained from our previous study and stored in liquid nitrogen until use [23]. Briefly, male Kunming mice were fed with a high-fat diet and induced with 80 mg/kg streptozotocin. The mice that met the criteria of T2DM (2 h-postprandial glucose level > 11.0 mM) were treated with 40 mg/kg/d of baicalein (Bal-LD), 160 of mg/kg/d baicalein (Bal-HD), and vehicle (DM) for 8 weeks. Normal mice treated with vehicle for 8 weeks were used as the control. The liver tissues each group were homogenized in lysis buffer containing protease inhibitor cocktail and 1 mM PMSF. The protein concentrations of the tissue lysate were then determined by the BCA assay. The corresponding immunoblot of PERK, Nrf2, HO-1, and CHOP was performed following the same procedure described above.

The cytoplasmic fraction and the nuclear fraction of HL-7702 cells and liver tissues were isolated using the cytoplasmic protein and nuclear protein isolation kit according to the user’s manual (Wanleibio Co., Ltd, Shenyang, China). The protein concentration of the samples was determined by the BCA assay according to the manufacturer’s instructions. The level of Nrf2 in the cytoplasmic fraction and the nuclear fraction was determined by immunoblotting according to the procedures described above using β-actin as the internal reference for cytoplasmic protein and Lamin B as the internal reference for nuclear protein.

### 4.8. Quantitative Real-Time Polymerase Chain Reaction (PCR) Analysis of mRNA

HL-7702 cells were seeded at a density of 2 × 10^5^ cells/well in 6-well plates and were allowed to adhere overnight. The cells were subjected to different treatments as described in Section 4.2. The total RNA from the HL-7702 cell was extracted with TRIzol reagent. The corresponding cDNA was synthesized with a cDNA synthesis kit according to the manufacturer’s instruction (Takara Co., Dalian, China). The relative levels of each mRNA transcripts to β-actin were determined by qRT-PCR using the SYBR pre-mixed system and specific primers. The primer sequences for *EIF2AK* (encoding PERK), *NFE2L2* (encoding Nrf2), *HMOX1* (encoding HO-1), *DDIT3* (encoding CHOP), and *ACTB* (encoding β-actin) were as follows: *EIF2AK*: 5′-GCACAGACGGGTCATTCCAC-3′ (forward) and 5′-TCCTATGTCGCCTTCACTCC-3′ (reverse); *NFE2L2*: 5′-GAGAATTCCTCCCAATTCAGC-3′ (forward) and 5′-TTTGGGAATGTGGGCAAC-3′ (reverse); *HMOX1*: 5′-CGTGCTCGCATGAACACTCT-3′ (forward) and 5′-GGCGGTCTTAGCCTCTTCTGT-3′ (reverse); *DDIT3*: 5′-TCTTCCTCCTCTTCCTCCTG-3′ (forward) and 5′-CACTCTTGACCCTGCTTCTC-3′ (reverse); *ACTB*: 5′-CCGTCTTCCCCTCCATCG-3′ (forward) and 5′-GTCCCAGTTGGTGACGATGC-3′ (reverse). Real-time qRT-PCR was performed by an ABI 7500 PCR system with a Power SYBR Green PCR Master Mix kit. The comparative cycle of threshold fluorescence (C_T_) method was used and the relative transcription level of the target gene was normalized to that of β-actin by the 2^−ΔΔCt^ method.

### 4.9. Statistic Analysis 

All the data were presented as the mean ± SD of at least three replicates. The statistical analysis was performed with SPSS version 17.0 (SPSS Inc., Chicago, IL, USA). One-way ANOVA followed by Student’s *t*-test was performed to evaluate the statistical significance of the data between each group and *p* < 0.05 was considered as statistically significant. 

## 5. Conclusions

The results of our study showed that baicalein alleviated the oxidative stress induced by a high level of glucose in HL-7702 cells. Furthermore, baicalein alleviated the level of apoptosis resulted from excessive oxidative stress. The anti-oxidative activity of baicalein is attributed to its regulation of the PERK/Nrf2 signaling pathway. Baicalein upregulated the level of Nrf2 and the oxidative stress-related protein HO-1 and downregulated the level of PERK and CHOP both in HL-7702 cells and in liver tissues from mice with T2DM. The majority of Nrf2 locates in the nucleus, and the nuclear Nrf2 level was significantly increased upon baicalein treatment both in vitro and in vivo. The change in the level of proteins was consistent with the changes in the expression level of the coding genes. Our results suggest that the alleviation of oxidative stress is one of the mechanisms contributing to the activity of baicalein in the prevention and treatment of diabetes. Baicalein is a promising candidate to be developed into a potential agent for the prevention and treatment of diabetes.

## References

[1] International Diabetes Federation http://www.diabetesatlas.org.

[2] Sun W., Zhang B., Zheng H., Zhuang C., Li X., Lu X., Quan C., Dong Y., Zheng Z., Xiu Z. (2017). Trivaric acid, a new inhibitor of PTP1b with potent beneficial effect on diabetes. Life. Sci..

[3] Saito T., Watanabe M., Nishida J., Izumi T., Omura M., Takagi T., Fukunaga R., Bandai Y., Tajima N., Nakamura Y. (2011). Lifestyle modification and prevention of type 2 diabetes in overweight Japanese with impaired fasting glucose levels. Arch. Intern. Med..

[4] Naudi A., Jove M., Ayala V., Cassanye A., Serrano J., Gonzalo H., Boada J., Prat J., Porterootin M., Pamplona R. (2012). Cellular dysfunction in diabetes as maladaptive response to mitochondrial oxidative stress. Exp. Diabetes. Res..

[5] Özcan U., Yilmaz E., Özcan L., Furuhashi M., Vaillancourt E., Smith R.O., Görgün C.Z., Hotamisligil G.S. (2006). Chemical chaperones reduce ER stress and restore glucose homeostasis in a mouse model of type 2 diabetes. Science.

[6] Zhang B., Chen Y., Shen Q., Liu G., Ye J., Sun G., Sun X. (2016). Myricitrin attenuates high glucose-induced apoptosis through activating Akt-Nrf2 signaling in H9c2 cardiomyocytes. Molecules.

[7] Singh R., Kishore L., Kaur N. (2014). Diabetic peripheral neuropathy: Current perspective and future directions. Pharmacol. Res..

[8] Bobrovnikovamarjon E., Pytel D., Riese M.J., Vaites L.P., Singh N., Koretzky G.A., Witze E.S., Diehl J.A. (2012). PERK utilizes intrinsic lipid kinase activity to generate phosphatidic acid, mediate Akt activation, and promote adipocyte differentiation. Mol. Cell. Biol..

[9] Hotamisligil G.S. (2010). Endoplasmic reticulum stress and the inflammatory basis of metabolic disease. Cell.

[10] Cullinan S.B., Zhang D., Hannink M., Arvisais E., Kaufman R.J., Diehl J.A. (2003). Nrf2 is a direct PERK substrate and effector of PERK-dependent cell survival. Mol. Cell. Biol..

[11] Fujiki T., Ando F., Murakami K., Isobe K., Mori T., Susa K., Nomura N., Sohara E., Rai T., Uchida S. (2019). Tolvaptan activates the Nrf2/HO-1 antioxidant pathway through PERK phosphorylation. Sci. Rep..

[12] Jyrkkänen H., Kuosmanen S., Heinäniemi M., Laitinen H., Kansanen E., Mella-Aho E., Leinonen H., Ylä-Herttuala S., Levonen A. (2011). Novel insights into the regulation of antioxidant-response-elementmediated gene expression by electrophiles: induction of the transcriptional repressor BACH1 by Nrf2. Biochem. J..

[13] Huang C., Wang J.J., Ma J.H., Jin C., Yu Q., Zhang S.X. (2015). Activation of the UPR protects against cigarette smoke-induced RPE apoptosis through up-regulation of Nrf2. J. Biol. Chem..

[14] Beara I.N., Lesjak M.M., Cetojević-Simin D.D., Marjanović Z.S., Ristić J.D., Mrkonjić Z.O., Mimica-Dukić N.M. (2014). Phenolic profile, antioxidant, anti-inflammatory and cytotoxic activities of black (*Tuber aestivum* Vittad.) and white (*Tuber magnatum* Pico) truffles. Food. Chem..

[15] You R., Pang Q., Li L. (2014). A metabolic phenotyping approach to characterize the effects of cantonese herbal tea on restraint stressed rats. Biol. Pharm. Bull..

[16] Bie B., Sun J., Guo Y., Li J., Jiang W., Yang J., Huang C., Li Z. (2017). Baicalein: A review of its anti-cancer effects and mechanisms in hepatocellular carcinoma. Biomed. Pharmacother..

[17] El-Bassossy H.M., Hassan N.A., Mahmoud M.F., Fahmy A. (2014). Baicalein protects against hypertension associated with diabetes: effect on vascular reactivity and stiffness. Phytomedicine.

[18] Sithisarn P., Michaelis M., Schubert-Zsilavecz M., Cinatl Jr J. (2013). Differential antiviral and anti-inflammatory mechanisms of the flavonoids biochanin A and baicalein in H5N1 influenza A virus-infected cells. Antivir. Res..

[19] Zhang B., Li X., Sun W., Xing Y., Xiu Z., Zhuang C., Dong Y. (2017). Dietary flavonoids and acarbose synergistically inhibit α-glucosidase and lower postprandial blood glucose. J. Agric. Food. Chem..

[20] Zhang B., Sang Y., Sun W., Yu H., Ma B., Xiu Z., Dong Y. (2017). Combination of flavonoids from *Oroxylum indicum* seed extracts and acarbose improves the inhibition of postprandial blood glucose: In vivo and in vitro study. Biomed. Pharmacother..

[21] Zhang B., Sun W., Yu N., Sun J., Yu X., Li X., Xing Y., Yan D., Ding Q., Xiu Z. (2018). Anti-diabetic effect of baicalein is associated with the modulation of gut microbiota in streptozotocin and high-fat-diet induced diabetic rats. J. Funct. Foods..

[22] Sun W., Sang Y., Zhang B., Yu X., Xu Q., Xiu Z., Dong Y. (2017). Synergistic effects of acarbose and an *Oroxylum indicum* seed extract in streptozotocin and high-fat-diet induced prediabetic mice. Biomed. Pharmacother..

[23] Sun W., Sun J., Zhang B., Xing Y., Yu X., Li X., Xiu Z., Dong Y. (2017). Baicalein improves insulin resistance via regulating SOCS3 and enhances the effect of acarbose on diabetes prevention. J. Funct. Foods..

[24] Pang M., Qu P., Gao C.L., Wang Z.L. (2012). Yessotoxin induces apoptosis in HL7702 human liver cells. Mol. Med. Rep..

[25] Chen T., Liu A.B., Sun S., Ajami N.J., Ross M.C., Wang H., Zhang L., Reuhl K., Kobayashi K., Onishi J.C. (2019). Green tea polyphenols modify the gut microbiome in *db/db* mice as co-abundance groups correlating with the blood glucose lowering effect. Mol. Nutr. Food. Res..

[26] Kang K.A., Zhang R., Piao M.J., Chae S., Kim H.S., Park J.H., Jung K.S., Hyun J.W. (2012). Baicalein inhibits oxidative stress-induced cellular damage via antioxidant effects. Toxicol. Ind. Health..

[27] Viduranga Y., Waisundara V.Y., Siu S.Y., Hsu A., Huang D., Tan B.K. (2011). Baicalin upregulates the genetic expression of antioxidant enzymes in Type-2 diabetic Goto-Kakizaki rats. Life. Sci..

[28] Naveenkumar C., Raghunandhakumar S., Asokkumar S., Devaki T. (2013). Baicalein abrogates reactive oxygen species (ROS)-mediated mitochondrial dysfunction during experimental pulmonary carcinogenesis *in vivo*. Basic. Clin. Pharmacol..

[29] Liu Y.W., Cheng Y.Q., Liu X.L., Hao Y.C., Li Y., Zhu X., Zhang F., Yin X.X. (2017). Mangiferin upregulates glyoxalase 1 through activation of Nrf2/ARE signaling in central neurons cultured with high glucose. Mol. Neurobiol..

[30] Lee I.K., Kang K.A., Zhang R., Kim B.J., Kang S.S., Hyun J.W. (2011). Mitochondria protection of baicalein against oxidative damage via induction of manganese superoxide dismutase. Environ. Toxicol. Phar..

[31] Wang T., Li Q., Bi K. (2018). Bioactive flavonoids in medicinal plants: Structure, activity and biological fate. Asian. J. Pharm. Sci..

[32] Zhang W., Hietakangas V., Wee S., Lim S.C., Gunaratne J., Cohen S.M. (2013). ER stress potentiates insulin resistance through PERK-mediated FOXO phosphorylation. Gene. Dev..

[33] Uruno A., Furusawa Y., Yagishita Y., Fukutomi T., Muramatsu H., Negishi T., Sugawara A., Kensler T.W., Yamamoto M. (2013). The Keap1-Nrf2 system prevents onset of diabetes mellitus. Mol. Cell. Biol..

